# Neuraminidase Gene Variations in Influenza A(H1N1)pdm09 Virus among Patients Admitted to Refferal Pulmonary Hospital, Tehran, Iran in 2009–2013

**Published:** 2017

**Authors:** Payam Momeni, Shabnam Abedin Dargoosh, Ali Akbar Sedehzadeh, Ghazal Bagheri, Mojgan Mohammadi, Leila Poosashkan, Afsaneh Sigaroodi, Makan Sadr, Seyed Alireza Nadji

**Affiliations:** 1 Virology Research Center, National Research Institute of Tuberculosis and Lung Diseases (NRITLD), Shahid Beheshti University of Medical Sciences, Tehran, Iran,; 2 Lung Transplantation Research Center, NRITLD, Shahid Beheshti University of Medical Sciences, Tehran, Iran,; 3 Islamic Azad University of Qom, Qom, Iran,; 4 Pediatric Respiratory Diseases Research Center, NRITLD, Shahid Beheshti University of Medical Sciences, Tehran, Iran,; 5 Tracheal Diseases Research Center, NRITLD, Shahid Beheshti University of Medical Sciences, Tehran, Iran

**Keywords:** Influenza A, H1N1, Neuraminidase gene, Epitope, Vaccine, Drug resistance mutation, Iran

## Abstract

**Background:**

Neuraminidase (NA) is one of the surface proteins of influenza A virus, which plays an important role in immunization against influenza infection and is recognized as an important therapeutic target. Genetic and antigenic changes and substitutions can influence the efficacy of vaccine and change viral sensitivity to NA inhibitors (NAIs). In this study, we performed phylogenetic and molecular analyses of NA changes in influenza A(H1N1)pdm09 virus, compared them with the corresponding vaccine strain, and examined drug resistance mutations in isolates from patients.

**Materials and Methods:**

The complete sequence of NA genes from 34 pandemic H1N1 isolates (identified in 2009–2010, 2010–2011, and 2013) was determined and analyzed both genetically and antigenically. The phylogenetic tree was plotted relative to the corresponding vaccine strain, using MEGA6 software package, based on the maximum likelihood method and JTT matrix (bootstrap value of 1000).

**Results:**

The phylogenetic analysis of pandemic isolates showed 31 amino acid substitutions in NA genes, compared to the vaccine strain. Some of these substitutions (N248D, V241I, N369K, N44S, and N200S) were important in terms of phylogenetic relationship, while the rest (D103N, V106I, R130T, N200S, G201E, and G414R) influenced the antigenic indices of B-cell epitopes. The catalytic sites, framework sites, and N-glycosylation remained unchanged in the studied samples. Meanwhile, H275Y substitution, related to oseltamivir resistance, was detected in 3 isolates. The average nucleotide identity of NAs with the corresponding vaccine strain was 99.415%, 98.607%, and 98.075% in 2009–2010, 2010–2011, and 2012–2013, respectively.

**Conclusion:**

In this study, we provided basic information on the genetic and antigenic changes of NA genes in influenza A(H1N1)pdm09 virus from patients in 3 different seasons in Tehran, Iran. Considering the viral NAI resistance and changes in NA gene sequences of the isolates in comparison with the vaccine strain, further studies should be performed to monitor genetic changes in Iran. Moreover, the efficacy of vaccines should be examined.

## INTRODUCTION

Influenza viruses are associated with significant morbidity and mortality and affect approximately 20% of the world’s population each year (1). In general, vaccination can provide optimal protection against influenza viruses. However, the level of protection by annual vaccination can be limited due to antigenic adaptation of circulating strains. In addition, the low level of vaccine coverage is another major issue in different communities (2).

Besides immunization, use of antiviral medicines, along with therapeutic or preventive strategies, can help prevent morbidity, mortality, and spread of influenza viruses (3). In this regard, phylogenetic analysis can provide valid information on the epidemiological development of influenza viruses. In fact, knowledge of the genetic and antigenic characteristics of these viruses can contribute to the development of laboratory-based diagnostic and surveillance methods (4). In general, continuous monitoring of circulating viruses is essential in evaluating the possible emergence of drug-resistant and viral strains, which may have the potential properties for vaccine development in the future (4).

Neuraminidase (NA) and hemagglutinin (HA) are among the most important transmembrane proteins of influenza virus and are strongly influenced by immunological pressure (1). The pressure, imposed by anti-NA antibodies, causes an antigenic drift in NA (5–7). In addition, NA is involved in the releasing of progeny virions, viral cell-to-cell spread, HA-mediated fusion, and effective virus proliferation (8–10).

Over the past decades, NA has been considered as a major drug target. Today, NA inhibitors (NAIs), such as oseltamivir, zanamivir, inhaled laninamivir, and peramivir, are available and used against influenza viruses, including A(H1N1)pdm09 virus (11). On the other hand, resistance mutations (H275Y, E119V/G, and I22V) have been reported in *in vivo* and *in vitro* models (12).

Since the emergence of influenza A(H1N1)pdm09 virus (13), many studies around the world have evaluated its genetic properties, drug resistance patterns, and virulent mutations. The Iranian population has been affected by the pandemic influenza virus since 2009. In our previous studies, we discussed the clinical features of influenza A(H1N1)pdm09 virus (14–16). In addition, we performed a study on the prevalence of oseltamivir resistance and H275Y mutations in pandemic H1N1 strains during 2009–2010 season, using real-time probe-based polymerase chain reaction (PCR) method (17).

With this background in mind, in the present study, we aimed to perform molecular and phylogenetic analyses of NA genes from 2009 pandemic H1N1 influenza viruses (identified in 2009–2010, 2010–2011, and 2012–2013) in patients referring to Masih Daneshvari Hospital, a specialized and referral center for lung diseases in Tehran, Iran. We also identified the genetic and antigenic changes with respect to the vaccine strain and monitored NAI resistance mutations.

## MATERIALS AND METHODS

### Samples

In this retrospective, cross sectional study, 34 respiratory samples, positive for influenza A(H1N1)pdm09 virus, were evaluated. The samples were collected randomly from the Virology Research Center, affiliated to the National Institute for Tuberculosis and Lung Diseases, Tehran, Iran between November 2009 and March 2013.

### Molecular analysis

RNA extraction from the samples was performed using the High-Pure Viral Nucleic Acid Kit (Roche, Germany). The presence of viral genomes was confirmed via real-time PCR method according to the World Health Organization (WHO)/Center for Disease Control (CDC) protocol (18). After confirming the presence of influenza A(H1N1)09pdm virus, NA genes were amplified using specific primers.

For assembling 1413-bp sequences in NA genes of pandemic strains, PCR was performed using 2 different primer sets, which amplified 956-bp (PN1F1; 5′- ATGAATCCAAACCAAAAGATAATAAC-3′ / PN1R1; 5′-ACTGCATATGTATCCTATCTG-3′) and 737-bp (PN1F2; 5′-GAACACAAGAGTCTGAATGTG-3′ / PN1R2; 5′- TTGTCAATGGTAAATGGCAAC-3′) segments.

### Phylogenetic analysis

The PCR products of NA genes were sequenced, using the BigDye Terminator v3.1 Cycle Sequencing Reaction Kit and ABI PRISM 3700 DNA Analyzer (Applied Biosystems). After editing and assembling with BioEdit software (version 7.0.5.3) (19), they were converted to amino acid sequences and aligned with A/California/ 7/2009 vaccine sequence (accession number, GQ377078). The phylogenetic tree was plotted using MEGA6 software package, based on the maximum likelihood method and JTT matrix (bootstrap value, 1000) (20). All the studies sequences were deposited in the GeneBank database under accession numbers MF196259-MF196293.

## RESULTS

In total, 193 out of 1092, 405, and 277 respiratory samples sent to the Virology Research Center during November 2009–March 2010, December 2010–March 2011, and November 2012–March 2013 were positive for A(H1N1)pdm09 virus, respectively (Table 1); finally, 34 samples were randomly selected among the positive samples. Based on the findings, the frequency of A(H1N1)pdm09 virus varied between 14.4% and 21.95% during the 3 evaluated periods.

**Table 1.** Real-Time PCR Assay data for detection of A(H1N1)09pdm on respiratory samples collected between 2009 and 2013.

**Year**	**Samples tested N**	**Positive for H1N1 pdm09 N (%)**
**2009–10**	410	90 (21.95)
**2010–11**	405	63 (15.5)
**2013**	277	40 (14.4)
**Total**	1092	193 (17.67)

**Figure 1.**
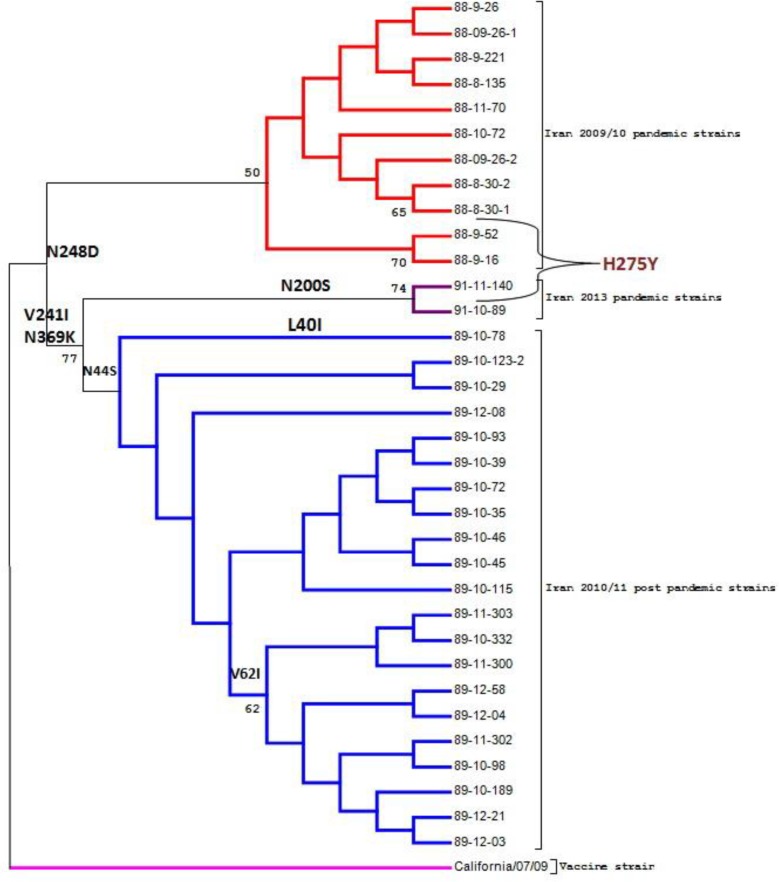
Maximum likelihood phylogenetic tree obtained with the alignment of NA sequences of pandemic H1N1 2009 viruses and Vaccine, using the JTT matrix, Mega 6 software. Bootstrap support values after 1000 pseudo-random replicates higher than 50% are shown in the corresponding nodes.

Figure 1 presents the phylogenic tree of NA sequence in the samples with respect to the corresponding vaccine strain. Based on the phylogenetic study of NA genes from pandemic influenza strains, 3 clades could be identified with respect to the branching pattern in the phylogenetic tree (Figure 1). In fact, A(H1N1)pdm09 viruses belonged to different periods and were completely separate in the phylogenetic tree.

Table 2 depicts the similarity of the studied strains to the vaccine strain, as well as the corresponding strain populations at amino acid and nucleotide levels. The measurements showed that the average nucleotide identity of the target strains with the corresponding vaccine strain had decreased from 2009 to 2013 at amino acid level (99.4% in 2009–2010 and 98.07% in 2012–2013). However, the average percentage of identity between the isolates had increased in each season (Table 2).

**Table 2.** Average NA sequence similarity within an individual and vaccine strain (A/California/7/2009)

**Items**	**2009–10 isolates**	**2010–11 isolates**	**2012–13 isolates**
**Within an individual at nucleotide level (%)**	94.154	96.567	96.150
**Within vaccine strain at amino acid level (%)**	99.415	98.607	98.075
**Within all individuals at amino acid level (%)**		98.802	

Table 3 presents a sequential analysis of amino acid sequence changes in NA genes of the studied strains in comparison with the vaccine strain. Overall, 31 amino acid substitutions were detected in the studied strains. The substitutions were as follows: I17V, A20T, N28D, L40I, L40V, N44S, V62I, S79P, V83M, S95N, D103N, V106I, C124R, R130T, P154S, N200S, G201E, N222D, V241I, N248D, R257K, V264A, K265P, H275Y, D284N, S285C, G320V, S334R, N369K, G414R, and T466P (Table 3).

**Table 3.** Amino acid variation in NA sequence during 2009–2010, 2010–2011 and 2013 comparison to Vaccine

**aa position**	**aa residue in vaccine^‡^**	**2009–2010**						**2010–2011**				**2013**	
		**Oct 2009**	**Nov 2009**	**Nov 2009**	**Dec 2009**	**Jan 2010**	**Feb 2010**	**Dec 2010**	**Jan 2011**	**Feb 2011**	**Mar 2011**	**Jan 2013**	**Feb 2013**
17	I	__	__	__	__	__	__	V	__	__	__	__	__
20	A	T	__	__	__	__	__	__	__	__	__	__	__
28	N	__	__	__	__	__	__	__	__	__	__	__	D
40	L	__	__	__	__	__	__	__	I	__	__	__	__
40	L	__	__	__	__	__	__	__	__	__	__	V	__
44	N	__	__	__	__	__	__	S	10/11 91%	S	5/6 83%	S	S
62	V	__	__	__	__	__	__	__	3/11 27%	I	4/6 67%	___	__
79	S	__	__	__	__	__	__	__	__	__	__	P	__
83	V	__	__	__	__	__	__	__	M	__	__	__	__
95	S	__	__	__	__	__	__	__	__	__	__	N	__
103	D	__	__	__	__	__	__	__	N	__	__	__	__
106	V	I	I	I	I	I	I	I	I	I	I	__	__
124	C	__	__	__	__	__	__	__	R	__	__	__	__
130	R	__	__	__	__	__	__	__	T	__	__	__	__
154	P	__	__	__	__	__	__	__	S	__	__	__	__
200	N	__	__	__	__	__	__	__	__	__	__	S	S
201	G	__	__	__	__	__	__	__	__	__	__	E	__
222	N	__	__	__	__	__	__	__	__	__	__	D	__
241	V	__	__	__	__	__	__	I	I	I	I	I	I
248	N	D	D	D	D	D	D	D	D	D	D	D	D
257	R	__	__	__	__	K	__	__	__	__	__	__	__
264	V	__	__	__	__	__	__	__	A	__	__	__	__
265	K	__	__	__	__	__	__	__	__	P	__	__	__
275^†^	H	__	__	Y	Y	__	__	__	__	__	__	__	Y
284	D	__	__	N	__	__	__	__	__	__	__	__	__
285	S	__	__	C	__	__	__	__	__	__	__	__	__
320	G	__	__	__	__	__	__	__	__	__	V	__	__
334	S	__	__	__	__	__	__	__	R	__	__	__	__
369	N	__	__	__	__	__	__	K	K	K	K	K	K
414	G	__	__	__	__	__	__	__	__	R	__	__	__
466	T	__	__	__	__	__	__	__	P	__	__	__	__

‡ A/California/7/2009

† H275Y; oseltamivir-resistant mutation

Table 4 presents the site and sequence of N-linked glycosylation in the studied samples. All 8 cases of N-linked glycolisation remained unchanged in the NA genes of pandemic strains (Table 4). In addition, Table 5 demonstrates relatively reserved epitopic sites of B cells in NA glycoproteins of influenza isolates in comparison with A/California/7/2009 vaccine strain. Mutations, including V106I, R130T, N200S, G201E, and G414R, were detected in the studied isolates, compared to the corresponding vaccine (Table 5). We used the study by Huang et al. (21) to evaluate B-cell epitopes, dependent on major histocompatibility complex (MHC) class I and II proteins.

**Table 4.** N-glycosylation sites in NA of pandemic H1N1 2009 viruses

**Item**	**Position**	**Sequence**
**1**	50	NQV
**2**	58	NNTW
**3**	63	NQTY
**4**	68	NISN
**5**	88	NSSL
**6**	146	NGTI
**7**	235	NGSC
**8**	386	NFS

**Table 5.** Comparison of conserve epitopes between NA sequences of studied subjects and Vaccine strain^‡^

**NA seq. epitope**		**Position**	**Possible B-cell epitope**	**Possible MHC I /MHC II alleles**	**abundance**
**Vaccine**	YSKDNSVRIGSKGDVFVIR	100–118	SIRIGSKGDV	A*0201,A*03/DRB1*0101,DRB1*0301,DRB1*0401	
**Iran str1**	YSKDNS I RIGSKGDVFVIR				most of isolates
**Iran str2**	YSKDNSVRIGSKGDVFVIR				2013; 2 isolates
**Iran str3**	YSKNNS I RIGSKGDVFVIR		DNSIRIGSKGDVFV	A*1101/DRB1*0701,DRB1*1101,DRB1*1501	2010/11;1 isolate
**Vaccine**	RTFFLTQGALLNDKHSN	130–146	FLTQGALLND	A*1101/DRB1*0701,DRB1*1101,DRB1*1501	
**Iran str1**	RTFFLTQGALLNDKHSN				most of isolates
**Iran str2**	TTFFLTQGALLNDKHSN				1 isolate
**Vaccine**	ISGPDNGAVAVLKYNGI	195–211	SGPDNGAVAVLKY	A*01,A*0201/DRB1*0301,DRB1*0401,DRB1*1501	
**Iran str1**	ISGPDNGAVAVLKYNGI		GPDNGAVAVL		most of isolates

‡ A/California/7/2009

## DISCUSSION

Two major preventive strategies against influenza virus include vaccination and antiviral medications. The effectiveness of influenza vaccine in preventing or reducing the severity of infection depends on the similarity of surface glycoproteins of vaccine strains to viral strains circulating in the community.

Influenza viruses are able to evade immune responses due to constant genetic changes in surface glycoproteins (including NA). Therefore, the antigenic changes of surface glycoproteins are reviewed annually to determine the optimal conditions for vaccine development (5, 6). Several studies have been conducted in Iran to evaluate the genetic and antigenic changes of seasonal influenza H1N1 and H3N2 viruses (22, 23). In this regard, a previous study examined the frequency of resistance to antiviral drugs in influenza A(H1N1)pdm09 strains (2012–2013) (24).

In the present study, NA genes of 34 influenza A(H1N1)pdm09 isolates were sequenced between November 2009 and March 2013 at Masih Daneshvari Hospital (a referal center for pulmonary diseases). Based on the phylogenic analysis, the isolates exhibited changes in comparison with the corresponding vaccine strain, and resistant mutants to NAIs were detected. The phylogenic study of NA genes indicated that the selected isolates were genetically related to the vaccine, although they showed continuous evolution from 2009 to 2013 (Figure 1). In fact, vaccine strain identity had decreased at nucleotide and amino acid levels over the years (Table 2).

Despite the decreased genetic identity of the isolates with the corresponding vaccine strain during 3 seasons, an increase was observed in the genetic relationship between the isolates in each season; this finding indicates the greater adaptation of pandemic influenza virus to the Iranian population over time (Table 2). In addition, Table 3 presents a sequential analysis of changes in NA genes relative to the vaccine strain at amino acid level.

Based on the present findings, although catalytic, framework, and glycosylation sites did not change in NA genes, 31 amino acid substitutions were detected in other regions. Overall, N44S, V62I, V106I, N200S, V241I, N248D, and N369K substitutions were most commonly detected (Table 3). Some other mutations have been also reported in other studies in Iran (24), which are of phylogenic importance.

The phylogenetic tree shows that the strains studied in each season constitute separate branches in the tree (Figure 1). Speciation of the isolates in different seasons may be due to mutations and substitutions, which are of phylogenic importance. N248D substitution distinguished all Iranian A(H1N1)pdm09 strains from the vaccine strain, as seen in all the isolates. Also, V241I and N369K mutations were observed in all 2010–2011 and 2012–2013 strains and distinguished them from 2009–2010 strains. N200S was only detected among 2012–2013 strains and helped distinguish 2012–2013 influenza A isolates. Meanwhile, N44S, V62I, and L40I mutations could internally distinguish 2010–2011 and 2012–2013 strains (Figure 1).

Studies around the world have introduced mutations involved in the development of NAI-resistant mutants with respect to the influenza virus subtype (11, 12). In the H1N1 subtype, the most important mutation is histidineto-tyrosine mutation at residue 275. This mutation makes the virus strongly resistant to oseltamivir. The present study showed that 3 isolates (2 isolates from 2009–2010 and 1 isolate from 2013) included H275Y substitution in NA genes (Figure 1, Table 3).

Two isolates in our previous study were detected via TaqMan real-time PCR (17). Although other studies performed in different countries have reported a significant increase in oseltamivir-resistant A(H1N1)pdm09 viruses (25–31), no cases have been detected in Iran (24). Also, other known mutations, which cause resistance to NAIs (such as D199N, E119V/G, and I223V/R), were not observed in this study.

Meanwhile, H275Y substitution, detected in this study, was of phylogenic importance and led to speciation of influenza strains in the phylogenetic tree (Figure 1). As stated earlier, in addition to enzymatic activity, NA exhibits antigenic properties. Therefore, in the study of influenza A(H1N1)pdm09 virus, antigenic changes in NA protein should be also investigated.

In the present study, examination of mutations in the epitopic regions with respect to the vaccine strain revealed changes in reserved B-cell-dependent antigenic parameters (Table 5). These changes included D103N and V106I substitutions in 100–118 amino acid residues, N200S and G201E substitutions in amino acid sequences at residues 195–211, R257K substitution at residues 250–258, and G414R substitution at residues 399–415. N200S substitution was observed in both 2012–2013 strains, whereas G201E was observed in only 1 strain from 2012–2013. The rest of the substitutions were sporadically detected in the epitopic regions.

The present study showed that NA gene changes have recently increased in A(H1N1)pdm09 viruses in Iran. Considering the amino acid changes in the epitopic sites of glycoproteins in A(H1N1)pdm09 virus (compared to the corresponding vaccine strain) and numerous infections among vaccinated physicians, health workers, and patients, further phylogenic studies should be performed on these viruses and efficacy of vaccines in Iran.

Overall, the available information on the prevalence and frequency of NAI-resistant variants is very limited in Iran. Given the increasing emergence of drug-resistant influenza viruses, we can limit the emergence of drug resistance through molecular monitoring, proper use of antiviral drugs, and development of laboratory-based diagnostic and surveillance strategies.
